# Testing for a treatment effect in a selected subgroup

**DOI:** 10.1177/09622802241277764

**Published:** 2024-09-25

**Authors:** Nigel Stallard

**Affiliations:** Warwick Clinical Trials Unit, Warwick Medical School, University of Warwick, Coventry, UK

**Keywords:** Adaptive enrichment design, family wise error rate control, hierarchical testing, linear regression, subgroup selection

## Abstract

There is a growing interest in clinical trials that investigate how patients may respond differently to an experimental treatment depending on the basis of some biomarker measured on a continuous scale, and in particular to identify some threshold value for the biomarker above which a positive treatment effect can be considered to have been demonstrated. This can be statistically challenging when the same data are used both to select the threshold and to test the treatment effect in the subpopulation that it defines. This paper describes a hierarchical testing framework to give familywise type I error rate control in this setting and proposes two specific tests that can be used within this framework. One, a simple test based on the estimated value from a linear regression model with treatment by biomarker interaction, is powerful but can lead to type I error rate inflation if the assumptions of the linear model are not met. The other is more robust to these assumptions, but can be slightly less powerful when the assumptions hold.

## 1. Introduction and motivation

Recent advances in the understanding of the heterogeneity of patients and disease types have led to an increasing desire to understand how patients may respond differently to a particular treatment. This in turn has led to development of a number of novel clinical trial approaches that attempt to identify a subgroup of patients that may benefit from an experimental therapy.^1,2^

One particular approach is the adaptive enrichment design.^3,4^ This is a two-stage design. In the first stage, patients are recruited from the whole population. Data from these patients are then used to select some subgroup of the population, which may be the whole population, in which a positive treatment effect is anticipated, with patients recruited from this subpopulation in the second stage. One such trial is described by Ho et al.^5^ At the end of the trial, the question of interest is whether the treatment can be shown to be effective in the subpopulation selected.

If this is a confirmatory, phase III, randomised controlled trial, as highlighted, for example, by Simon,^6^ the final analysis of the trial is challenging. It is often considered desirable to conduct a hypothesis test of the treatment effect in the selected subgroup, but if the final analysis uses data from both stages the fact that stage 1 data are used both to select the subpopulation to be included in the second stage and to assess the effectiveness of the treatment in that subpopulation can lead to inflation of the type I error rate. In order to control the type I error rate it has been suggested that only the data from the second stage might be used in the final analysis^7^ or that data from all patients in the first stage are combined with those in the second stage irrespective of whether or not they are in the selected subpopulation,^4^but such approaches are generally inefficient.^8^

While there has been some work on methods that enable use of the first stage data in the final analysis and control the type I error rate, these have predominantly been in the case in which the subpopulation is defined on the basis of a single binary biomarker so that in the second stage recruitment is either from the whole population or restricted to a predefined subgroup including only biomarker positive patients.^9–13^

There has been more limited work on the setting in which the biomarker is continuous. In this case, while some authors assume that this continuous biomarker divides the patients into two distinct groups with different levels of treatment effect,^14^, it is more common to assume that the biomarker has a monotonic effect on the treatment effect, often with the direction of this assumed, so that, for example, the treatment effect is assumed to be non-decreasing with increasing biomarker values. Selection of the subpopulation thus corresponds to identification of a threshold level for the biomarker so that the subgroup is comprised of all patients with biomarker levels above this threshold. Lin et al.^8^ consider this problem in the setting of a single-arm trial, and propose selection of the subgroup that maximises the standardised average subpopulation effect relative to a specified value, which may be a historical treatment effect. Stallard^15^ considers the setting of a two-arm study with the subgroup selected to maximise one of the measures including the test statistic comparing the treatment groups in the selected subgroup and the estimated treatment difference in the subgroup. Selection based on maximisation of a test statistic such as the estimated treatment difference can, however, lead to selection of small subgroups when the treatment effect is increasing with the biomarker.

Frieri et al.^16^ extend the setting of Lin et al.^8^ to that of a comparative trial, with patients randomised to receive either an experimental treatment or a standard treatment considered as a control. Like Lin et al.,^8^ they assume that the biomarker and the response follow a bivariate normal distribution, so that the expected response is linearly related to the biomarker level, and propose selection of the subpopulation of patients with biomarker levels such that the expected response on the experimental treatment exceeds that on the control treatment by some specified amount. Frieri et al., like Wang et al.,^17^ who consider time to event data, and Baldi Antognini et al.,^18^who consider design issues, focus primarily on estimation of a threshold level for the continuous biomarker above which the treatment effect is positive. The final analysis they propose is, however, based only on the stage 2 patients from the selected subgroup, so does not need to take account of the data-dependent treatment selection but does not use all available data. The focus of the current paper is more on hypothesis testing when data from both stages are used, the aim being to identify a subpopulation in which the null hypothesis that the treatment is ineffective can be rejected whilst allowing for the multiple testing that arises from the same data being used to both identify the subpopulation and conduct the test.

## 2. Setting and notation

Since, as outlined above, the statistical challenges arise from the use of the same data for both subgroup selection and hypothesis testing within the selected subgroup, we will focus specifically on the analysis of data from stage one. Data from stage two can be added, for example, via a combination test,^19^ as described in more detail in the Discussion section below. We thus consider a single-stage study in which a continuous biomarker is used to identify a subpopulation with the treatment effect tested in that subpopulation. Like Lin et al.^8^ and Frieri et al.,^16^ we will specifically consider the setting of a continuous response that can be assumed to be normally distributed, though we will make no similar assumption about the distribution of the biomarker.

In detail, suppose n patients are assigned to two groups, with $t_{i}$ indicating group membership for patient i , i=1,…,n , with $t_{i}=0$ for patients receiving a control treatment and $t_{i}=1$ for patients receiving an experimental treatment. Let $n_{1}=t_{1}+\cdots +t_{n}$ and $n_{0}=n-n_{1}$ denote the numbers of patients in treatment groups 1 and 0, respectively. Treatment allocation is typically assigned at random or using a randomly permuted blocks design to ensure that $n_{0}=n_{1}=n/2$ .

Let $x_{i}$ denote the biomarker value for patient i , i=1,…,n . We will condition these values, considering them fixed. Without loss of generality, assume $x_{1}\geq x_{2}\geq \cdots \geq x_{n}$ .

Let $Y_{i}$ denote the response for patient i , with $y_{i}$ denoting the observed value of $Y_{i}$ , i=1,…,n , and $Y_{i}$ normally distributed and related to $x_{i}$ and $t_{i}$ via a linear regression model with main effects and interaction. That is

(1) $$Y_{i}=\alpha_{0}+\beta_{0}x_{i}+\alpha t_{i}+\beta t_{i}x_{i}+\varepsilon_{i},i=1,\ldots ,n$$

with $\varepsilon_{i}$ independent $N(0,\sigma^{2})$ for some unknown σ .

We will assume that larger values of $Y_{i}$ are more desirable, and will further assume that it is known, or can be assumed a priori, that β≥0 ; that is, if the biomarker has a predictive effect then it is in the direction such that treatment effects are larger for larger biomarker values.

## 3. Closed testing procedure for strong familywise error rate control

Let θ(x) denote the treatment effect at biomarker level x , so that, from equation (1), we have θ(x)=α+βx , and write $\theta_{k}$ for $\theta (x_{k})$ . We wish to test the family of null hypotheses $H_{k}:\theta_{k}\leq 0$ for k=1,…,n . Note that, since $x_{1}\geq \cdots \geq x_{n}$ , the assumption β≥0 means that $\theta_{1}\geq \cdots \geq \theta_{n}$ . Hence $H_{1}\subseteq \cdots \subseteq H_{n}$ and $H_{k}$ implies θ(x)≤0 for all $x\leq x_{k}$ . Conversely, if we reject $H_{k}$ and conclude $\theta_{k}>0$ , this implies we may conclude that θ(x)>0 for all $x\geq x_{k}$ . Rejecting $H_{k}$ thus leads to the conclusion that the treatment is effective for patients with biomarker level $x_{k}$ or larger.

Since $H_{1}\subseteq \cdots \subseteq H_{n}$ , the set $\left\{H_{k};k=1,\ldots ,n\right\}$ is closed under intersection. Applying the closed testing procedure,^20^ for given k , the familywise error rate (FWER) is controlled in the strong sense by a procedure under which $H_{k}$ can be rejected if all intersection hypotheses that are subsets of $H_{k}$ can be rejected at the nominal level. In this case, this corresponds to the rejection of $H_{1},\ldots ,H_{k}$ .

**Figure 1.**
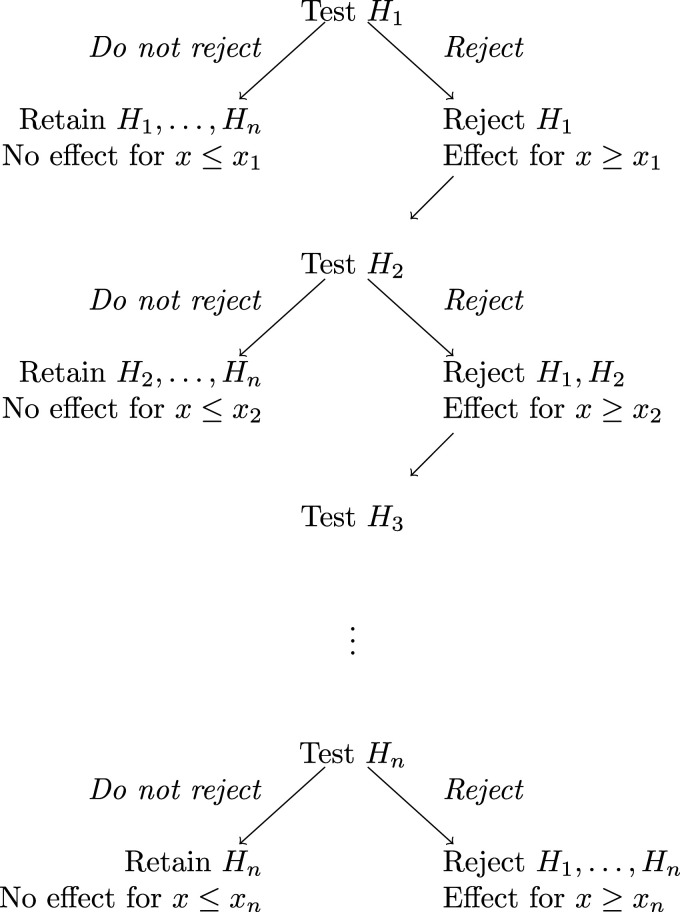
The hierarchical testing procedure for the family of hypotheses $\left\{H_{k},k=1,\ldots ,n\right\}$ .

The FWER is thus strongly controlled by the hierarchical testing procedure illustrated in Figure 1, in which we test each $H_{k}$ at the nominal level, starting with k=1 . If $H_{k}$ is not rejected at the nominal level, we do not reject any of $H_{k},\ldots ,H_{n}$ and conduct no further tests. If $H_{k}$ is rejected at the nominal level, we reject $H_{1},\ldots ,H_{k}$ , concluding that θ(x)>0 for all $x\geq x_{k}$ , and proceed to step k+1 .

## 4. A simple regression-based test of $H_{k}$

In order to apply the hierarchical testing procedure just described, a test of $H_{k}$ is required. As noted above, $H_{k}$ implies $\theta (x_{k})\leq 0$ and hence θ(x)≤0 for all $x\leq x_{k}$ , thus in particular for $x_{k},\ldots ,x_{n}$ . A test of $H_{k}$ can thus be based on data $(x_{i},y_{i},t_{i}),i=k,\ldots ,n$ . Although this test is not based on the entire data set, and so may lack power for larger k , omitting data $(x_{i},y_{i},t_{i}),i=1,\ldots ,k-1$ means that rejection of $H_{k}$ , and a conclusion that the treatment is effective at biomarker level $x_{k}$ , cannot arise due to observed data indicating a large treatment effect at higher biomarker levels.

In order to test $H_{k}$ based on the data $(x_{i},y_{i},t_{i}),i=k,\ldots ,n,$ we can fit the linear model given by (1) to the data $(x_{i},y_{i},t_{i}),i=k,\ldots ,n$ , noting that this requires k≤n−3 for parameter estimates to be identifiable. Fitting the model provides estimates of the treatment and treatment by biomarker interaction effects, ${\hat{\alpha }}_{k}$ and ${\hat{\beta }}_{k}$ , together with their estimated variances and covariance. The estimated treatment effect at a biomarker value $x_{k}$ is then $\hat{\theta }(x_{k})={\hat{\alpha }}_{k}+{\hat{\beta }}_{k}x_{k}$ with expected value $E(\hat{\theta }(x_{k}))=\alpha_{k}+x_{k}\beta_{k}$ and variance $\mathrm{var}(\hat{\theta }(x_{k}))=\mathrm{var}({\hat{\alpha }}_{k})+2x_{k}\mathrm{cov}({\hat{\alpha }}_{k},{\hat{\beta }}_{k})+x_{k}^{2}\mathrm{var}({\hat{\beta }}_{k}),$ where estimates and their variances can be obtained using standard software, for example, using the lm and vcov commands in R,^21^or via expressions in, for example, Madsen and Thyregod.^22^. Since $E(\hat{\theta }(x_{k}))=\theta (x_{k})$ , we can test $H_{k}$ using $Z_{k}=\hat{\theta }(x_{k})/\sqrt{\mathrm{var}(\hat{\theta }(x_{k}))}$ , which under $H_{k}$ has an asymptotic distribution that is stochastically no larger than a N(0,1) -distributed random variable.

If ${\hat{\beta }}_{k}>0$ , so that the data support a treatment effect that increases with x , a (one-sided) p -value for the test $H_{k}$ can thus be obtained as

(2) $$p_{k}=1-\Phi (Z_{k})=1-\Phi \left\{\frac{\hat{\theta }(x_{k})}{\sqrt{\mathrm{var}(\hat{\theta }(x_{k}))}}\right\}.$$

If ${\hat{\beta }}_{k}<0$ , indicating a treatment effect that decreases with x , we do not wish to conclude that θ(x)>0 for all $x\geq x_{k}$ , so set $p_{k}=1$ .

Similarly, for k>n−3 , when it is impossible to fit the linear model (1) to the data $(x_{i},y_{i},t_{i}),i=k,\ldots ,n$ , we set $p_{k}=1$ and fail to reject $H_{k}$ . In practice, this is unlikely to be of concern when n is of reasonable size as it is not desirable to conclude that the treatment is effective for a very small subset of the population.

## 5. A more robust test of $H_{k}$

As the estimate $\hat{\theta }(x_{k})$ on which the p -value given by (2) is based is taken directly from the linear model (1), it might be anticipated that the test based on this p -value would be sensitive to departure from the linearity assumed, and it is shown below that this can be the case, particularly when the true treatment effect θ(x) is increasing and concave. An alternative, more robust, test of $H_{k}$ is thus also proposed.

As in the simpler test above, the test of $H_{k}$ is based on data $(x_{i},y_{i},t_{i}),i=k,\ldots ,n$ , that is, data with $x\leq x_{k}$ . With this procedure, we first identify a value $x_{k}^{*}$ such that a positive treatment effect is anticipated for $x\geq x_{k}^{*}$ , then use data with $x_{k}^{*}\leq x\leq x_{k}$ to test for a treatment effect after adjusting for x to test the hypothesis $H_{k}$ .

**Figure 2.**
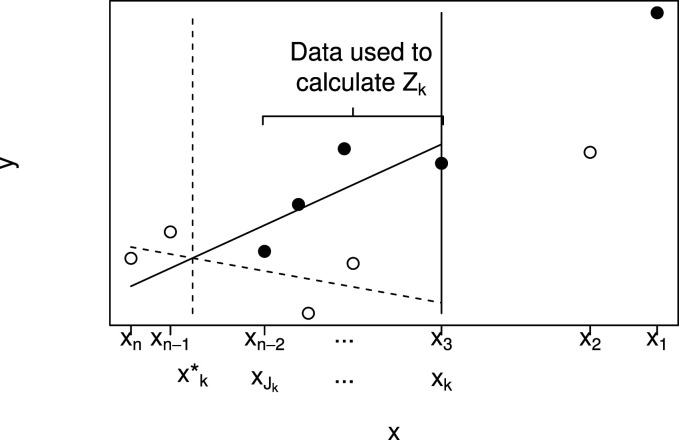
Plot showing $y_{i}$ plotted against $x_{i}$ for a single small data set using solid points for $t_{i}=1$ and hollow points for $t_{i}=0$ to illustrate notation and data used to test $H_{k}$ (here with k=3 ). A linear model with treatment by biomarker interaction is fitted to data for patients i=k,…,n and the value of x at which the lines cross is denoted by $x_{k}^{*}$ . The value $J_{k}$ is then the largest i with $x_{i}\geq x^{*}$ . Data for patients $i=k,\ldots ,J_{k}$ are used to calculate the test statistic, which will be denoted $Z_{k}^{*}$ .

The procedure is illustrated by Figure 2, which shows a plot of $y_{1},\ldots ,y_{n}$ against $x_{1}\geq \cdots \geq x_{n}$ for a particular data set. Solid points indicate $t_{i}=1$ and hollow points $t_{i}=0$ . In order to test $H_{k}$ , data $(x_{i},y_{i},t_{i}),i=k,\ldots ,n$ , are used. This is illustrated in the figure for k=3 , so that data given by points to the right of the solid vertical line at $x_{3}$ are not used.

We first fit the linear model (1), with main effects and interaction, to the data $(x_{i},y_{i},t_{i}),i=k,\ldots ,n$ , again requiring that k≤n−3 , to obtain estimates ${\hat{\alpha }}_{k}$ , ${\hat{\beta }}_{k}$ with estimated treatment effect at a biomarker value x given by $\hat{\theta }(x)={\hat{\alpha }}_{k}+{\hat{\beta }}_{k}x$ . If ${\hat{\beta }}_{k}>0$ , so that the data support a treatment effect that increases with x , the estimated treatment effect, $\hat{\theta }(x)$ is positive for all $x\geq x_{k}^{*}$ where $x_{k}^{*}=-{\hat{\alpha }}_{k}/{\hat{\beta }}_{k}$ .

The fitted lines from model (1) are shown in Figure 2 by the dashed line (group 0) and solid line (group 1). The point at which these lines cross is $x_{k}^{*}$ , shown by the vertical dashed line. Given $x_{k}^{*}$ , we define $J_{k}=\arg\;\max\{x_{j}|x_{j}\geq x_{k}^{*}\}$ , with $J_{k}=0$ if $\left\{x_{j}|x_{j}\geq x_{k}^{*}\right\}$ is empty, so that $x_{J_{k}}$ is the smallest $x_{j}$ at least as large as $x_{k}^{*}$ .

If ${\hat{\beta }}_{k}>0$ and $J_{k}\geq k$ , the treatment effect in the range $x_{J_{k}}\leq x\leq x_{k}$ adjusted for x can be estimated by fitting a linear model with main effects of treatment and biomarker but no interaction to data $(x_{i},y_{i},t_{i}),i=k,\ldots ,J_{k}$ assuming $k\leq J_{k}-2$ . A test statistic, $Z_{k}^{*}$ , for $H_{k}$ can then be obtained as the test for a treatment effect adjusted for x from this model. The data used to obtain fit $Z_{k}^{*}$ lie between the vertical dashed and solid lines in Figure 2. If ${\hat{\beta }}_{k}<0$ or $J_{k}<k$ , the data do not indicate that there are any $x<x_{k}$ with θ(x)>0 and we set $Z_{k}^{*}=-\infty$ .

In order to obtain a p -value, $p_{k}^{*}$ , for the test of $H_{k}$ , the observed value of the test statistic $Z_{k}^{*}$ can be compared to its null distribution. This distribution is derived in the following subsection.

### 5.1. Null distribution of $Z_{k}^{*}$ , the test statistic for a treatment effect in the selected subgroup

In order to base a test of $Z_{k}^{*}$ , we require its null distribution.

We start by giving a derivation of the distribution of $Z_{k}^{*}$ under the simple null hypothesis α=β=0 .

As described above and illustrated in Figure 2, let $x_{k}^{*}=-{\hat{\alpha }}_{k}/{\hat{\beta }}_{k}$ and $J_{k}=\arg\;\min\{x_{j}|x_{j}\geq x_{k}^{*}\}$ . We wish to obtain the distribution of $Z_{k}^{*}$ , that is, specifically to obtain $Pr(Z_{k}^{*}\geq c).$

We have

$$\begin{array}{r} Pr(Z_{k}^{*}\geq c)=\sum_{j=k}^{n}Pr(Z_{k}^{*}\leq c,J_{k}=j) \end{array}$$

which, since $Z_{k}^{*}=-\infty$ if ${\hat{\beta }}_{k}\leq 0$ , means that

$$\begin{array}{r} Pr(Z_{k}^{*}\geq c)=\sum_{j=k}^{n}Pr(Z_{k}^{*}\leq c,J_{k}=j,{\hat{\beta }}_{k}>0) \end{array}$$

Now, for $J_{k}=j$ , we must have $x_{k}^{*}\in (x_{j+1},x_{j}]$ , that is, $x_{j+1}<-{\hat{\alpha }}_{k}/{\hat{\beta }}_{k}\leq x_{j}.$ If ${\hat{\beta }}_{k}>0$ , that is, $-{\hat{\alpha }}_{k}-x_{j+1}{\hat{\beta }}_{k}>0$ and ${\hat{\alpha }}_{k}+x_{j}{\hat{\beta }}_{k}\geq 0,$ so that

(3) $$Pr(Z_{k}^{*}\geq c)=\sum_{j=k}^{n}Pr(Z_{k}^{*}\geq c,-{\hat{\alpha }}_{k}-x_{j+1}{\hat{\beta }}_{k}>0,{\hat{\alpha }}_{k}+x_{j}{\hat{\beta }}_{k}\geq 0,{\hat{\beta }}_{k}>0)$$

Since $Z_{k}^{*},-{\hat{\alpha }}_{k}-x_{j+1}{\hat{\beta }}_{k},{\hat{\alpha }}_{k}+x_{j}{\hat{\beta }}_{k}$ and ${\hat{\beta }}_{k}$ are all linear combinations of coefficients from a linear model involving data from patients i=k,…,n , we have

$$\begin{array}{r} {\left(Z_{k}^{*},-{\hat{\alpha }}_{k}-x_{j+1}{\hat{\beta }}_{k},{\hat{\alpha }}_{k}+x_{j}{\hat{\beta }}_{k},{\hat{\beta }}_{k}\right)}^{\prime }=MY_{k} \end{array}$$

for some 4×(n−k) matrix M , with $Y_{k}=(y_{k},\ldots ,y_{n}{)}^{\prime }$ , so that

(4) $${\left(Z_{k}^{*},-{\hat{\alpha }}_{k}-x_{j+1}{\hat{\beta }}_{k},{\hat{\alpha }}_{k}+x_{j}{\hat{\beta }}_{k},{\hat{\beta }}_{k}\right)}^{\prime }∼N\left(ME\left(Y_{k}\right),M\mathrm{var}\left(Y_{k}\right)M^{\prime }\right)$$

(see Appendix 1 in the Supplemental material for details).

If $\sigma^{2}$ were known, the probability

(5) $$Pr(Z_{k}^{*}\geq c,-{\hat{\alpha }}_{k}-x_{j+1}{\hat{\beta }}_{k}>0,{\hat{\alpha }}_{k}+x_{j}{\hat{\beta }}_{k}\geq 0,{\hat{\beta }}_{k}>0)$$

could thus be given by a multivariate normal tail area.

Since the terms on the left-hand side, all come from linear models adjusting for the biomarker levels, they do not depend on $\alpha_{0}$ or $\beta_{0}$ , so these may be set to 0 in the distribution (4). Hence under the null hypothesis α=β=0 , we can take $E(Y_{k})=0$ , the zero vector, so that the four elements of the vector on the left-hand side of equation (4) all have mean 0.

Thus, the value of $Z_{k}^{*}$ is invariant to scaling of $Y_{k}$ , since it is divided by σ . Scaling of $Y_{k}$ will lead to scaling of $-{\hat{\alpha }}_{k}-x_{j+1}{\hat{\beta }}_{k}$ , ${\hat{\alpha }}_{k}+x_{j}{\hat{\beta }}_{k}$ and ${\hat{\beta }}_{k}$ , but since in the probability given in equation (5) these are compared with their mean values 0, the probability will remain unchanged. We may thus scale each $Y_{i}$ to have unit variance so that equation (4) becomes

(6) $${\left(Z_{k}^{*},-{\hat{\alpha }}_{k}-x_{j+1}{\hat{\beta }}_{k},{\hat{\alpha }}_{k}+x_{j}{\hat{\beta }}_{k},{\hat{\beta }}_{k}\right)}^{\prime }∼N\left(0,MM^{\prime }\right)$$

Denoting by $\bar{\Phi }(b,\mu ,\Sigma )$ the complementary distribution function giving the probability $Pr(X_{i}\geq b_{i}\text{ for all }i)$ when X has a multivariate normal distribution with mean μ and variance–covariance matrix Σ , the probability given by equation (5) is equal to $\bar{\Phi }((c,0,0,0{)}^{\prime },0,MM^{\prime }).$ This then enables calculation of $Pr(Z_{k}^{*}\geq c)$ given by equation (3) as required.

In practice, since σ is unknown, the estimated value from the linear regression (1) could be used. This estimate could be obtained separately for each $H_{k}$ using data $(x_{i},y_{i},t_{i}),i=k,\ldots ,n$ . However, since the estimate may be poorly estimated when the sample size used to fit the models is small, as will be the case when testing $H_{k}$ for k large or when $J_{k}$ is close to k , an alternative is to use an estimate of σ obtained from fitting the model (1) to the whole data set when calculating $Z_{k}^{*}$ and the corresponding p -value. This will provide a more precise estimate if (1) holds with $\mathrm{var}(\varepsilon_{i})=\sigma^{2}$ for all i .

The p -value given by (3) was based on the distribution of $Z_{k}^{*}$ under the simple null hypothesis α=β=0 . Supplemental Appendix 2 shows that this distribution is stochastically larger than that under any α,β such that β≥0 and $\alpha +\beta x_{k}\leq 0$ , and hence α+βx≤0 , that is, θ(x)≤0 , for all $x\leq x_{k}$ , so that a test based on the p -value obtained is thus conservative under any other null scenario.

The test statistic $Z_{k}^{*}$ is obtained by using the data in the range $x_{J_{k}}\leq x\leq x_{k}$ to test for a treatment effect adjusting for x . Alternative tests could fit a model to these data with a treatment effect but without adjusting for x , or adjusting for both x and an interaction between x and treatment, could also be used, with the distribution of the test statistic obtained in a similar way to that of $Z_{k}^{*}$ .

## 6. Simulation study

### 6.1. Comparison of operating characteristics

A simulation study was conducted to assess the properties of the testing procedures described above.

Taking n=80 , for each of 10,000 simulated data sets, values $t_{1},\ldots ,t_{n}$ were sampled at random without replacement from the set with 40 elements equal to 0 and 40 elements equal to 1 so that $n_{0}=n_{1}=40$ and values $x_{1},\ldots ,x_{n}$ were simulated from a normal distribution with mean 0 and variance 1 and ordered such that $x_{1}\geq \cdots \geq x_{n}$ . Values $y_{1},\ldots ,y_{n}$ were then simulated from the model

(7) $$Y_{i}∼N(\alpha_{0}+\beta_{0}x_{i}+\alpha t_{i}+\beta t_{i}x_{i},1)$$

with α=0 and β taken to be 0, 0.5, and 1 to illustrate a range of treatment by biomarker interaction effects. As the models used adjust for the biomarker, the results of the hypothesis tests are invariant to $\alpha_{0}$ and $\beta_{0}$ and these were arbitrarily set to zero. Hypotheses $H_{1},\ldots ,H_{n}$ were tested at FWER 0.025 using the tests based on $Z_{k}$ and $Z_{k}^{*}$ as described above.

For β=0 , a type I error corresponds to rejection of any $H_{k}$ while for β>0 , since α=0 so that θ(x)≤0 if and only if x<0 , a type I error corresponds to rejection of any $H_{k}$ with $x_{k}<0$ . The number of simulations leading to a type I error was recorded. For β>0 , the number of simulations leading to rejection of $H_{k}$ for each $x_{k}>0$ was also recorded as a measure of power. Note that the hierarchical testing procedure used, as illustrated in Figure 1, means that this number is increasing in $x_{k}$ (and decreasing in k ).

In order to provide a comparison with the approaches described above, the data were also used to test $H_{1},\ldots ,H_{k}$ using an approach based on that described by Stallard.^15^ In this approach, a nominal test of $H_{k}$ was conducted by fitting the linear model to test for a treatment effect adjusted for a biomarker effect to data sets $(x_{i},y_{i},t_{i}),i=k,\ldots ,j$ for j=k+2,…,n and taking the test statistic to be the largest observed from the (n−k−1) test statistics obtained. A p -value for the test based on this maximum can be obtained as described by Stallard^15^ and the hierarchical testing procedure applied as above.

**Figure 3.**
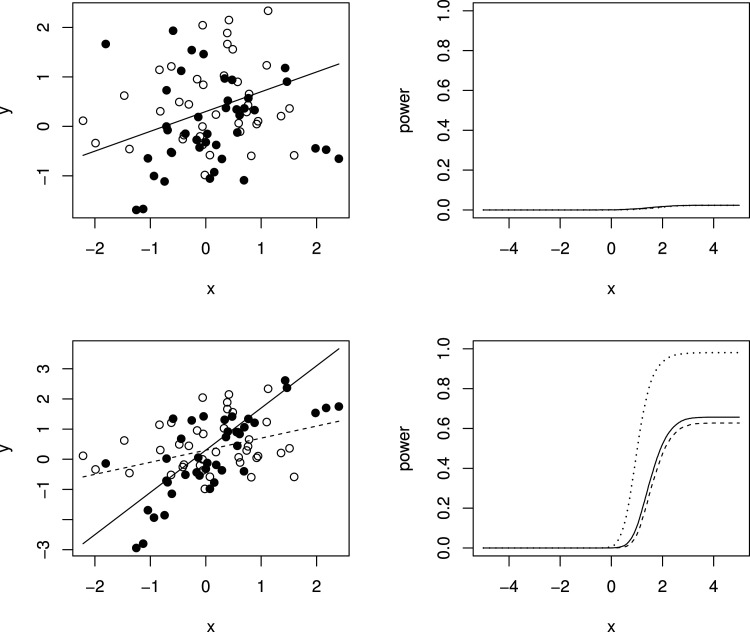
Simulation models and example data sets and simulation results for β=0 (upper panels) and β=1 (lower panels). Left-hand panels show simulation models and one simulated data set for t=0 (dashed line gives expected values and hollow points give simulated values) and t=1 (solid line gives expected values and solid points give simulated values). Right-hand panels show simulated probability from 10,000 simulations per scenario of rejecting $H_{k}$ plotted against $x_{k}$ for tests as in Stallard^15^ (dashed line) and using $Z_{k}$ (dotted line) and $Z_{k}^{*}$ (solid line). Probabilities are type I error rates for β=0 and for $x_{k}\leq 0$ when β=1 and powers for β=1 and $x_{k}>0$ .

Simulation results are given in Figure 3 and Table 1. The left-hand panels of Figure 3 show the expected values under the simulation models for t=0 (dotted line) and t=1 (solid line) for β equal to 0 in the top row, so that the dashed and solid lines coincide, and to 1 in the second row, together with one simulated data set in each case with hollow points for t=0 and solid points for t=1 given as for illustration of the variability of the data around the expected values. The right-hand panel shows, for the same values of β , the estimated probability of rejecting $H_{k}$ plotted against $x_{k}$ for k=1,…,n using the tests described above based on $Z_{k}$ and $Z_{k}^{*}$ and using the test proposed by Stallard.^15^. This probability corresponds to the type I error rate in the upper plot, and to the type I error for x≤0 and the power for x>0 in the lower plot. The overall type I error and power for $H_{1}$ for β∈{0,0.5,1} in each case are given in Table 1.

**Table 1.** Estimated familywise type I error rate (FWER) and power from simulation study (estimates based on 10,000 simulations).

	Test based on $Z_{k}$		Test based on $Z_{k}^{*}$		Test based on largest test statistic	
β	FWER^a^	Power^b^	FWER^a^	Power^b^	FWER^a^	Power^b^
0	0.0233	–	0.0234	–	0.0236	–
0.5	0.0024	0.5159	0.0013	0.2042	0.0005	0.2029
1	0.0070	0.9738	0.0006	0.6563	0.0001	0.6273

^a^FWER is the probability of rejecting any $H_{k}$ if β=0 or any $H_{k}$ with $x_{k}\leq 0$ if β>0 .

^b^Power is probability of rejecting any $H_{k}$ with $x_{k}>0$ if β>0 .

It can be seen that all of the proposed procedures control the familywise type I error rate at the 0.025 level as required. For β=0, the FWER is close to the nominal level. For larger β>0 , the type I error rate reported is the probability of rejecting any $H_{k}$ with $x_{k}\leq 0$ . Since in this case the treatment effect $\theta (x_{k})$ decreases with $x_{k}$ , for small $x_{k}$ values we can have $\theta (x_{k})$ well below 0, leading to conservatism in the test.

It is also evident that the test using $Z_{k}$ is more powerful than that using $Z_{k}^{*}$ , and that this is slightly more powerful than the test proposed by Stallard^15^ that chooses the value of $x^{*}$ that gives the largest test statistic.

The probability of rejecting $H_{k}$ for specified $x_{k}$ shown in the lower right-hand plot shows the power to detect a treatment effect at different biomarker levels, x . As the hierarchical testing procedure ensures that rejection of $H_{k}$ leads to rejection of $H_{j}$ for all j≤k , this power increases with x , reducing towards the type I error rate as x approaches zero from above, for any testing method. This seems reasonable for the model considered, for which the treatment effect θ(x) is reducing towards zero at x=0 . Since in the simulations the $x_{k}$ values are simulated from a N(0,1) distribution, many of the $x_{k}$ values are close to 0, so that even for $x_{k}>0$ many true treatment effect values, $\theta_{k}$ , are small. The power to reject $H_{k}$ is thus low for many k , leading to low true discovery rates, which, for β=1 are, respectively, estimated from the simulations to be 35%, 13% and 10% for the methods using $Z_{k}$ , $Z_{k}^{*}$ and the approach of Stallard.

### 6.2. Robustness to departures from the linear model

Both of the tests for $H_{k}$ described above are based on the linear model (1). If the assumed model is incorrect, neither of the test statistics $Z_{k}$ nor $Z_{k}^{*}$ will follow the distributions obtained and the test may be inaccurate.

In order to assess the robustness of the methods to departures from (1) a number of simulations were conducted with data simulated from other models. In order to assess potential type I error rate inflation, simulations were conducted under null scenarios in which $E(Y_{i}|x_{i},t_{i}=1)\leq E(Y_{i}|x_{i},t_{i}=0)\leq 0$ for all $x_{1},\ldots ,x_{n}$ .

Writing θ(x) for $E(Y_{i}|x_{i},t_{i}=1)-E(Y_{i}|x_{i},t_{i}=0)$ , the first sets of simulations were conducted with data simulated from models with θ(x)=0 for all x but with $E(Y_{i}|x_{i},t_{i}=1)$ not linearly related to x , that is, with the biomarker X having no predictive effect but a non-linear prognostic effect. Additional sets of simulations were conducted with $E(Y_{i}|x_{i},t_{i}=0)$ constant and θ(x) non-linearly increasing in x but with simulated values of X restricted to ensure that θ(x)≤0 for all simulated X values. These models correspond to the biomarker X having no prognostic effect but a non-linear predictive effect such that the treatment is not effective for any patients. The first two sets of simulation models thus all correspond to null models in which there is no positive treatment effect at any biomarker level. A third set of simulations were conducted with $E(Y_{i}|x_{i},t_{i}=0)$ constant and θ(x) non-linearly increasing in x but with θ(x)>0 for x>0 and θ(x)<0 for x<0 .

The simulation models are given in Table 2, and are also illustrated, along with one example simulated data set in each case, in the left-hand panels of Figures A1 to A3 given in Appendix 3 in the Supplemental material, which are analogous to Figure 3 above. In each case, data were simulated with $n_{0}=n_{1}=40$ , X∼N(0,1) and Y normal with variance 1 and mean related to X according to the expression given in the table.

**Table 2.** Non-linear models used for simulations reported in Table 3 and in Supplemental Appendix 3.

Description	$E(Y_{i}|x_{i},t_{i})$
Models with prognostic biomarker effect	
Step function	$-1+2I(x_{i}>0)$
Concave	$2+0.03(x_{i}-4{)}^{3}$
Convex	$-2+0.03(x_{i}+4{)}^{3}$
Models with predictive biomarker effect (positive treatment effect for no x values)	
Concave^a^	$2+0.03(x_{i}-4{)}^{3}I(t_{i}=1)$
Convex^b^	$8.3+(0.03(x_{i}+4{)}^{3}-10.3)I(t_{i}=1)$
Models with predictive biomarker effect (positive treatment effect for x>0 )	
Step function	$-1+(2+I(t_{i}=1))I(x_{i}>0)$
Concave	$2+0.03((x_{i}-4{)}^{3}+176)I(t_{i}=1)$
Convex	$8.3+0.03((x_{i}+4{)}^{3}-64)I(t_{i}=1)$

^a^ $x_{i}\leq 4$ to ensure $E(Y_{i}|x_{i},t_{i}=1)<E(Y_{i}|x_{i},t_{i}=0)$ .

^b^ $x_{i}\leq 3$ to ensure $E(Y_{i}|x_{i},t_{i}=1)<E(Y_{i}|x_{i},t_{i}=0)$ .

Estimated type I error rates based on 10,000 simulations under each model are given in Table 3 and are also given in Figures A1 to A3 in Appendix 3 in the Supplemental material. It can be seen that there is a slight increase in the type I error rate above the nominal level for tests based on $Z_{k}$ and $Z_{k}^{*}$ when there is a convex prognostic effect of x and for the test based on $Z_{k}$ when there is a concave prognostic effect. Particularly notable, however, is the very large increase in the type I error for the test based on $Z_{k}$ when there is a concave predictive effect of x . The tests proposed by Stallard^15^ that do not use a linear model control the type I error rate in all cases, but are again conservative, unsurprisingly particularly when θ(x)<0 for some values of x .

**Table 3.** Simulation models and estimated familywise type I error rates from 10 000 simulations to assess robustness to departures from the linear model.

	Test based	Test based	Test based on largest
Model	on $Z_{k}$	on $Z_{k}^{*}$	test statistic
Models with prognostic biomarker effect			
	Familywise type I error rate		
Step function	0.0261	0.0226	0.0122
Concave	0.0365	0.0085	0.0110
Convex	0.0840	0.0309	0.0124
Models with predictive biomarker effect (positive treatment effect for no x values)			
	Familywise type I error rate		
Concave^a^	0.4015	0.0078	0.0029
Convex^b^	0.0000	0.0000	0.0000
Models with predictive biomarker effect (positive treatment effect for x>0 )			
	Familywise type I error rate		
Step function	0.0055	0.0025	0.0018
Concave	0.0172	0.0001	0.0006
Convex	0.0049	0.000	0.0000
	Power (percentage hypotheses correctly rejected)		
Step function	0.5005 (20%)	0.5269 (19%)	0.5645 (21%)
Concave	0.8585 (32%)	0.2298 (4%)	0.1259 (2%)
Convex	0.9945 (45%))	0.9712 (23%)	0.9869 (22%)

^a^ $x_{i}\leq 4$ to ensure $E(Y_{i}|x_{i},t_{i}=1)<E(Y_{i}|x_{i},t_{i}=0)$ .

^b^ $x_{i}\leq 3$ to ensure $E(Y_{i}|x_{i},t_{i}=1)<E(Y_{i}|x_{i},t_{i}=0)$ .

For models with a positive treatment effect for some x values, estimated power, again based on 10,000 simulations under each model, is also given in Table 3 together with estimated true rejection rates. It can be seen that the power of all methods depends on the true model for the data. In particular, the estimated power is largest for the convex model for which the true treatment effect, θ(x) , is large for larger x , and smallest for the concave model for which θ(x) is relatively small for all x , and particularly for smaller positive x . The test based on $Z_{k}$ has the highest power, consistent with the increased type I error rate, though, for the step function model, all three methods have similar power.

## 7. Example: Analysis of data from an Alzheimer’s disease study

Schnell et al.^23^ report an analysis of data from a trial of an experimental therapy for Alzheimer’s disease. They compare patients receiving low-dose treatment with those receiving a placebo in terms of their change in cognitive impairment, with positive values indicating an improvement, that is, a reduction in cognitive impairment, identifying a positive treatment by age interaction indicating that the treatment is more effective for older patients.

**Figure 4.**
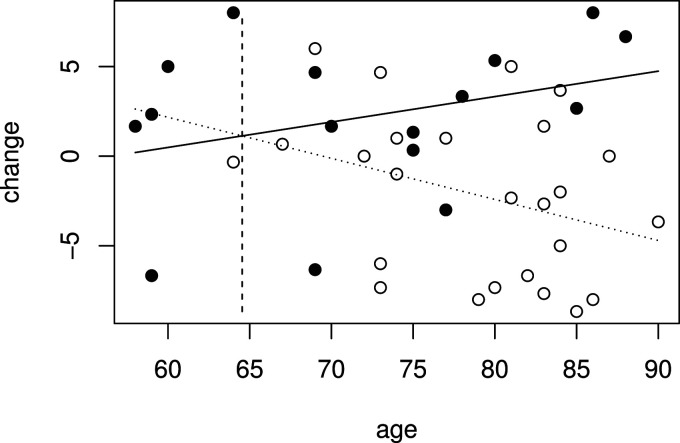
Improvement in cognitive impairment and age for patients in low dose (solid points) and placebo groups (hollow points) in Alzheimer’s disease study.

The data from 41 patients, with 16 receiving low-dose treatment and 25 receiving placebo, are available from Schnell et al.^23^ and are shown in Figure 4, where the outcome is plotted against age with placebo patients represented by a hollow circle and low-dose patients by a solid circle.

Figure 4 also shows fitted values for the linear model given by (1) that includes terms for treatment, age and a treatment by age interaction fitted to the whole data set. This can be used to test the null hypothesis $H_{1}$ . From the linear model, the estimate $\hat{\beta }$ is equal to 0.371, so is greater than zero, indicating that the treatment effect is increasing with age, and the estimated treatment effect at the largest observed age, $x_{1}=90$ , is 9.44 with standard error 2.86, so that $Z_{1}=3.30$ , corresponding to a p -value of 0.0005. Fitted treatment effects are positive for patients of age $x^{*}=-\hat{\alpha }/\hat{\beta }=64.5$ and above. The value of $x^{*}$ is shown by the vertical dashed line in Figure 4. Using data from patients with $x_{i}\geq x^{*}$ to construct $Z_{1}^{*}$ gives $Z_{1}^{*}=2.60$ . A p -value can be found as described above, and in this case is equal to 0.0036.

In order to control the FWER for testing of hypotheses $H_{1},\ldots ,H_{n}$ , the hierarchical procedure described above and illustrated in Figure 1 is applied. Results of tests for hypotheses $H_{k},k=1,2,\ldots$ based on $Z_{k}$ and $Z_{k}^{*}$ are given in Table 4. In this data set, $x_{4}=x_{5}$ and $x_{6}=x_{7}$ , so that the hypotheses $H_{4}$ and $H_{5}$ and the hypotheses $H_{6}$ and $H_{7}$ are identical and are tested using the same data $(x_{i},y_{i},t_{i}),i=4,\ldots ,n$ and $(x_{i},y_{i},t_{i}),i=6,\ldots ,n$ respectively. The results for the tests of each of these pairs of hypotheses are thus given in the same line in Table 4.

**Table 4.** Results of hypothesis tests for $H_{k}:\theta (x)\leq 0\text{ for }x\geq x_{k}$ for k=1,…,8 in Alzheimer’s disease study data shown in Figure 4.

k	$x_{k}$	$Z_{k}$	$p_{k}$	$x_{k}^{*}$	$Z_{k}^{*}$	$p_{k}^{*}$
1	90	3.30	0.0005	64.5	2.60	0.0036
2	88	3.30	0.0005	64.9	2.54	0.0045
3	87	2.81	0.0025	64.8	2.10	0.0144
4, 5	86	2.98	0.0014	65.8	2.12	0.0141
6, 7	85	2.03	0.0212	64.9	1.45	0.0650
8	84	1.49	0.0687	–	–	–

Using the test based on $p_{k}$ , the hierarchical procedure leads to rejection of hypotheses $H_{1},\ldots ,H_{7}$ at the one-sided 0.025 level, and to retention of hypotheses $H_{8},\ldots ,H_{n}$ without any further testing, since the p -value for the test of $H_{8}$ is 0.0687, and thus exceeds 0.025. As $x_{7}=85$ , we can conclude that there is a significant effect of treatment for patients aged 85 and above.

For the test based on $p_{k}^{*}$ hypotheses $H_{1},\ldots ,H_{5}$ are rejected at the one-sided 0.025 level with $H_{6},\ldots ,H_{n}$ retained as $p_{6}^{*}=0.0650$ exceeds 0.02. In this case, it would be concluded that there is a significant treatment effect for patients aged $x_{5}=86$ and above.

It is worth noting that the smallest $x_{k}$ for which $H_{k}$ is rejected, in this case, $x_{7}=85$ or $x_{4}=86$ , are considerably larger than $x_{1}^{*}$ , the estimated value above which the treatment effect is positive, which in this case is equal to 64.5 as noted above. This reflects the fact that the subgroup for which there is evidence of benefit is smaller than that for which there is an indication of benefit.^23^ While this is a particular feature of a setting such as this when the sample size is low, in general, since we desire to have a low probability of rejecting $H_{k}$ for $x_{k}$ with $\theta (x_{k})=0$ , the power will be low for values of x just above this even for larger sample sizes. This is analogous to the setting in a simple comparison of groups where the treatment effect estimate may be positive but not statistically significantly larger than 0.

## 8. Discussion

This paper has presented an approach for hypothesis testing to compare two groups in a subpopulation selected on the basis of some continuous biomarker when the same data are used for the selection and the hypothesis test. Although motivated by the setting of a clinical trial with a predictive biomarker, the method could be applied in other settings in which a subgroup of a data set is selected.

In the biomarker setting, as described in the Introduction, a popular approach is to use an adaptive enrichment design in which recruitment is restricted to the selected subpopulation in the second stage of a two-stage trial. The method proposed could be used in such a setting by using a combination test to combine evidence from both stages for tests of each hypothesis within the hierarchical testing framework described. Stage two patients could either be recruited from a subpopulation defined to be patients with any biomarker level with a positive estimated effect based on the analysis of the stage one data or from the subpopulation with biomarker values corresponding to a sufficiently small p -value based on the stage one data.

Like the approaches of Lin et al.^8^ and Frieri et al.,^16^ the proposed method is based on a model in which a continuous response is related to the biomarker values via a normal linear model. Unlike these authors, however, we do not assume bivariate normality for the biomarker and response values. Rather, we condition the biomarker values, so that the methods make no assumption about their distribution in the population. The method could thus also be used in settings where biomarker levels are chosen by design or sampled from a restricted range within the population. In the simulations and example presented above, the values $x_{1},\ldots ,x_{n}$ at which the null hypotheses are tested are the observed biomarker levels for the n patients in the study, with these patients sampled at random from the population under investigation. An alternative approach might be to either stratify sampling or to use test hypotheses at biomarker levels that do not correspond to all observed values, for example corresponding to certain quantiles of the anticipated or empirical distribution of biomarker levels observed. Such an approach could also ensure that the number of patients with data used for tests of $H_{k}$ for larger k was not too small.

In contrast to Frieri et al. and Baldi Antognini et al.^18^ our focus is not primarily on estimation of the biomarker level above which the experimental treatment is more effective than the control, but on identification of a subpopulation in which a positive treatment effect can be demonstrated. It should also be noted that we do not take as our parameter of interest the average treatment effect in the identified subpopulation. If the treatment effect depends on a continuous biomarker, a group selected with a positive average treatment effect will include patients with biomarker levels such that the expected treatment effect is negative. Our test based on $\theta_{k}$ , the smallest treatment effect in the selected group, will avoid this.

The methods proposed are based on the assumption that response and biomarker values are linearly related, with the usual assumptions of a normal linear regression model holding. This is a common assumption in this setting and may often be reasonable, but should be checked using the data. The second method presented, based on the test statistic $Z_{k}^{*}$ , appears to be more robust, as indicated in the simulation study presented, but can still lead to type I error rate inflation. The alternative method proposed by Stallard^15^ does not rely on linear model assumptions for control of the family wise type I error rate. This could thus be a more appropriate method to use if it was considered that the assumption of a linear relationship between response and biomarkers might not hold. The simulation studies reported above indicate, however, that the gain in robustness of the method of Stallard^15^ comes at a cost of a loss in power relative to the approach proposed above. In practice, it is recommended that simulation studies are used to assess both the type I error rate and the power of the approach selected for a range of possible scenarios and sample sizes to ensure that the method is sufficiently robust and the study appropriately powered.

Other tests, including those not based on a linear model or including higher order polynomial terms to allow for a non-linear biomarker effect, could also be used within the hierarchical testing framework described and might have attractive properties in terms of type I error rate control and power. Simulation studies could also be used to assess the power of alternative testing methods under a range of treatment effect scenarios considered to be likely. An assumption that the treatment effect θ(x) is increasing in the biomarker is required, though this may well be considered reasonable in many contexts where a predictive biomarker effect is hypothesised.

It is also assumed that the biomarker levels are measured without error. In some settings, error in these measurements might be considered sufficiently small that it can be ignored and conventional linear regression models, as proposed above, can be used. If this is not considered reasonable, alternative error-in-variables methods could be used to replace the tests proposed above to allow for the uncertainty in the true biomarker levels,^24^ though this would still assume that the subsets used in the hierarchical testing approach are correct.

The methods proposed have been developed and applied with a continuous, normally distributed response. The hierarchical testing framework could be used with other settings such as those with binary or time-to-event responses given suitable tests of the individual hypotheses $H_{k}$ . Extensions of the first method proposed might be possible based on estimated treatment effects from a logistic or Cox regression model, but a derivation of the distribution of the test statistic using an extension of the second method might be more challenging.

## Supplemental Material

sj-pdf-1-smm-10.1177_09622802241277764 - Supplemental material for Testing for a treatment effect in a selected subgroup Supplemental material, sj-pdf-1-smm-10.1177_09622802241277764 for Testing for a treatment effect in a selected subgroup by Nigel Stallard in Statistical Methods in Medical Research
