# Supplementary material for: Testing for a treatment effect in a selected subgroup
Source: Stat Methods Med Res. 2024 Sep 25;33(11-12):1967–78. doi: 10.1177/09622802241277764 (PMC11577705; doi:10.1177/09622802241277764)
Supplement: sj-pdf-1-smm-10.1177_09622802241277764 - Supplemental material for Testing for a treatment effect in a selected subgroup [file sj-pdf-1-smm-10.1177_09622802241277764.pdf]

TESTING FOR A TREATMENT EFFECT IN A SELECTED SUBGROUP

NIGEL STALLARD

Warwick Clinical Trials Unit, University of Warwick, UK

n.stallard@warwick.ac.uk

SUPPLEMENTARY MATERIAL

Appendix 1

1.1 Models and notation

The method proposed will involve fitting linear models with main effects of the biomarker and treatment, with and without an interaction term, to sets of data of the form $(x_i, y_i, t_i), i = i_0, \ldots, i_1$ for $1 \leq i_0 < i_1 \leq n$. Notation associated with these models that will be used below is first defined.

Model 1: adjusting for x with interaction term

Model 1 is the linear model with interaction term given by equation (1) in the main paper, only now with $i = i_0, \ldots, i_1$, in which

$$E(Y_i) = \alpha_0 + \beta_0 x_i + \alpha t_i + \beta t_i x_i.$$

Let $\mathbf{D}^{[i_0:i_1]}$ denote the design matrix given by

$$\mathbf{D}^{[i_0:i_1]} = \begin{pmatrix} 1 & x_{i_0} & t_{i_0} & x_{i_0}t_{i_0} \\ 1 & x_{i_0+1} & t_{i_0+1} & x_{i_0+1}t_{i_0+1} \\ \vdots & \vdots & \vdots & \vdots \\ 1 & x_{i_1} & t_{i_1} & x_{i_1}t_{i_1} \end{pmatrix} \tag{A.1}$$

and let $\mathbf{Y}^{[i_0:i_1]} = (Y_{i_0}, \ldots, Y_{i_1})'$.

Denoting the rows of the matrix $\left(\mathbf{D}^{[i_0:i_1]\,\prime}\mathbf{D}^{[i_0:i_1]}\right)^{-1}\mathbf{D}^{[i_0:i_1]\,\prime}$ by $\mathbf{a}_0^{[i_0:i_1]}$, $\mathbf{b}_0^{[i_0:i_1]}$, $\mathbf{a}^{[i_0:i_1]}$ and $\mathbf{b}^{[i_0:i_1]}$ respectively, estimates of $\alpha_0, \alpha, \beta_0$ and β are given by

$$\begin{aligned} \hat{\alpha}_0^{[i_0:i_1]} &= \mathbf{a}_0^{[i_0:i_1]\,\prime}\mathbf{Y}^{[i_0:i_1]} \\ \hat{\beta}_0^{[i_0:i_1]} &= \mathbf{b}_0^{[i_0:i_1]\,\prime}\mathbf{Y}^{[i_0:i_1]} \\ \hat{\alpha}^{[i_0:i_1]} &= \mathbf{a}^{[i_0:i_1]\,\prime}\mathbf{Y}^{[i_0:i_1]} \\ \hat{\beta}^{[i_0:i_1]} &= \mathbf{b}^{[i_0:i_1]\,\prime}\mathbf{Y}^{[i_0:i_1]}. \end{aligned}$$

Model 2: adjusting for x without interaction term

Model 2 is the linear model with main effects of treatment and biomarker without an interaction term in which

$$E(Y_i) = \alpha_0 + \beta_0 x_i + \alpha t_i.$$

Let $\mathbf{X}^{[i_0,i_1]}$ denote the design matrix for this model using data $(x_i, y_i, t_i), i = i_0, \ldots, i_1$, that is the matrix comprising the first three columns of $\mathbf{D}^{[i_0:i_1]}$ given by equation (8).

Denoting respectively the third row and the element in the third row and column of the matrix $\left(\mathbf{X}^{[i_0:i_1]\,\prime}\mathbf{X}^{[i_0:i_1]}\right)^{-1}\mathbf{X}^{[i_0:i_1]\,\prime}$ by $\mathbf{a}^{[i_0:i_1]}$ and $a^{[k,j]}$, the pa-

rameter α is estimated by

$$\hat{\alpha}^{[i_0:i_1]} = \mathbf{a}^{[i_0:i_1]\,\prime}\,\mathbf{Y}^{[i_0:i_1]}$$

with, for known σ, variance given by

$$var(\hat{\alpha}^{[k,j]}) = a^{[k,j]}\sigma^2.$$

Let

$$Z^{[i_0,i_1]} = \frac{\hat{\alpha}^{[i_0,i_1]}}{se(\hat{\alpha}^{[i_0,i_1]})},$$

be a test statistic to test for a treatment effect adjusted for X on the basis of data from patients $i = i_0, \ldots, i_1$, then

$$Z^{[i_0,i_1]} = \mathbf{z}^{[i_0,i_1]\,\prime}\,\mathbf{Y}^{[i_0,i_1]}$$

where

$$\mathbf{z}^{[i_0,i_1]} = \frac{\mathbf{a}^{[i_0,i_1]}}{\sigma a^{[i_0,i_1]}}.$$

Since

$$Z_k^* = \mathbf{z}^{[k:j]\,\prime}\,\mathbf{Y}^{[k:j]},$$

$$-\hat{\alpha}_k - x_{j+1}\hat{\beta}_k = \left(-\mathbf{a}^{[k:n]} - x_{j+1}\mathbf{b}^{[k:n]}\right)'\,\mathbf{Y}^{[k:j]},$$

$$\hat{\alpha}_k + x_j\hat{\beta}_k = \left(\mathbf{a}^{[k:n]} + x_j\mathbf{b}^{[k:n]}\right)'\,\mathbf{Y}^{[k:j]}$$

and

$$\hat{\beta}_k = \mathbf{a}^{[k:n]\,\prime}\,\mathbf{Y}^{[k:j]},$$

if we write $\mathbf{M}^{[k:n]}$ for the matrix with rows equal to $\mathbf{z}^{[k:j]\,\prime}$, $\left(-\mathbf{a}^{[k:n]} - x_{j+1}\mathbf{b}^{[k:n]}\right)'$,

$\left(\mathbf{a}^{[k:n]} + x_j \mathbf{b}^{[k:n]}\right)'$ and $\mathbf{a}^{[k:n]\,\prime}$, we have

$$\begin{pmatrix} Z_k^* \\ -\hat{\alpha}_k - x_{j+1}\hat{\beta}_k \\ \hat{\alpha}_k + x_j\hat{\beta}_k \\ \hat{\beta}_k \end{pmatrix} = \mathbf{M}^{[k:n]}\mathbf{Y}^{[k:j]}$$

so that

$$\begin{pmatrix} Z_k^* \\ -\hat{\alpha}_k - x_{j+1}\hat{\beta}_k \\ \hat{\alpha}_k + x_j\hat{\beta}_k \\ \hat{\beta}_k \end{pmatrix} \sim N\left(\mathbf{M}^{[k:n]} E\left(\mathbf{Y}^{[k:j]}\right), \mathbf{M}^{[k:n]\,\prime}\, var\left(\mathbf{Y}^{[k:j]}\right)\mathbf{M}^{[k:n]}\right). \tag{A.2}$$

Appendix 2

As above, for given $j \geq k$, let Z_k denote the test statistic for testing for a treatment effect from a linear model including treatment and biomarker fitted to data $(y_i, x_i, t_i), i = k, \ldots, j$.

To test the H_k we will use $Z^{[k:J_k]}$, where $J_k = \arg\max\{x_i \mid x_i \geq x^{[k:n]}\}$ with $x_k^* = -\hat{\alpha}_k/\hat{\beta}_k$.

Assuming $Y_1, \ldots, Y_n$ are given by (1), the distributions of the random variables $Z^{[k:j]}$, x_k^*, J_k and $Z^{[k:J_k]}$ depend on the true values of the regression parameters α and β. To make this explicit, we will write $Z^{[k:j]}(\alpha, \beta)$ and so on.

In order to calculate a p-value based on the observed value of $Z^{[k:J_k]}$ we require its distribution under the hypothesis H_k. The distribution under $\alpha = \beta = 0$ is derived above. The following result shows that this stochastically dominates the distribution in the null region H_k as required to provide a valid test.

Theorem

For given $k = 1, \ldots, n$, sufficiently small for the models using data $(x_i, y_i, t_i), i = k, \ldots, n$ to be fit, let $Z^{[k:J_k]}$ be as defined above with the distribution of $Y_1, \ldots, Y_n$ as given by (1). Suppose that $\beta \geq 0$ and α and β are such that

$$\alpha + \beta x \leq 0 \text{ for all } x \leq x_k \tag{A.3}$$

that is are such that H_k holds.

Then, writing $A \leq_{st} B$ to denote (first order) stochastic dominance of a random variable A by B, we have

$$Z^{[k:J_k]}(\alpha, \beta) \leq_{st} Z^{[k:J_k]}(0, 0).$$

Proof

Writing $\theta(x, \alpha, \beta)$ for $\alpha + \beta x$, from (A.3) we have $\theta(x, \alpha, \beta) \leq \theta(x, 0, 0) = 0$, so that, for given $j \geq k$ we have

$$Z^{[k:j]}(\alpha, \beta) \leq_{st} Z^{[k:j]}(0, 0). \tag{A.4}$$

As, for $\theta(x, 0, 0) = 0$, and so does not depend on x, and $\theta(x, \alpha, \beta)$ is increasing in x so that $\theta(x_k, \alpha, \beta) \geq \cdots \theta(x_{j-1}, \alpha, \beta) \geq \theta(x_j, \alpha, \beta)$, for given $j \geq k + 1$ we have

$$Z^{[k:j]}(0, 0) \leq_{st} Z^{[k:j-1]}(0, 0) \tag{A.5}$$

and

$$Z^{[k:j]}(\alpha, \beta) \leq_{st} Z^{[k:j-1]}(\alpha, \beta). \tag{A.6}$$

Let c be some constant with $c \leq \max\{x_k, \ldots, x_n\}$, that is $c \leq x_k$. Since

$$x_k^*(\alpha, \beta) = \frac{-\hat{\beta}_k(\alpha, \beta)}{-\hat{\beta}_k(\alpha, \beta)}$$

we have

$$x_k^*(\alpha, \beta) \geq c$$

if and only if $\hat{\beta}_k(\alpha, \beta) \geq 0$ and

$$-\hat{\alpha}_k(\alpha, \beta) \geq c\hat{\beta}_k(\alpha, \beta),$$

that is if and only if

$$-\hat{\alpha}_k(\alpha, \beta) - c\hat{\beta}_k(\alpha, \beta) \geq 0. \tag{A.7}$$

Similarly,

$$x_k^*(0, 0) \geq c$$

if and only if

$$-\hat{\alpha}_k(0,0) - c\hat{\beta}_k(0,0) \geq 0. \tag{A.8}$$

Since the random variables on the left hand sides of (A.7) and (A.8) are both normal with equal variance, since this depends only on $x_k, \ldots, x_n$ and σ^2, and with means $\alpha - c\beta$ and 0 respectively, with $\alpha - c\beta \leq 0$ by (A.3), we have

$$x_k^*(\alpha, \beta) \geq_{st} x_k^*(0,0)$$

and hence, since J_k decreases as x_k^* increases,

$$J_k(\alpha, \beta) \leq_{st} J_k(0,0). \tag{A.9}$$

The stated result then follows from equations (A.4), (A.5), (A.6) and (A.9) via Theorem 2.2.8 of Belzunce *et al.* (2016).

References

Belzunce, F., Martinéz-Riquelme, C. and Mulero, J. (2016) *An introduction to stochastic orders.* Elsevier Academic Press.

Appendix 3

Additional simulation results from the models given in Table 2 of the main paper (which is reproduced as Table A.1 here) are given in Figures A.1 to A.3.

Table A.1: Non-linear simulation models used to give results illustrated in Figures A.1, A.2 and A.3

Description	$E(Y_i \mid x_i, t_i)$
Models with prognostic biomarker effect	
Step function	$-1 + 2I(x_i > 0)$
Concave	$2 + 0.03(x_i - 4)^3$
Convex	$-2 + 0.03(x_i + 4)^3$
Models with predictive biomarker effect (positive treatment effect for no x values)	
Concave[a]	$2 + 0.03(x_i - 4)^3 I(t_i = 1)$
Convex[b]	$8.3 + (-10.3 + 0.03(x_i + 4)^3) I(t_i = 1)$
	[a] $x_i \leq 4$ to ensure $E(Y_i \mid x_i, t_i = 1) < E(Y_i \mid x_i, t_i = 0)$
	[b] $x_i \leq 3$ to ensure $E(Y_i \mid x_i, t_i = 1) < E(Y_i \mid x_i, t_i = 0)$
Models with predictive biomarker effect (positive treatment effect for $x > 0$)	
Step function	$-1 + (2 + I(t_i = 1)) I(x_i > 0)$
Concave	$2 + 0.03((x_i - 4)^3 + 176) I(t_i = 1)$
Convex	$8.3 + 0.03((x_i + 4)^3 - 64) I(t_i = 1)$

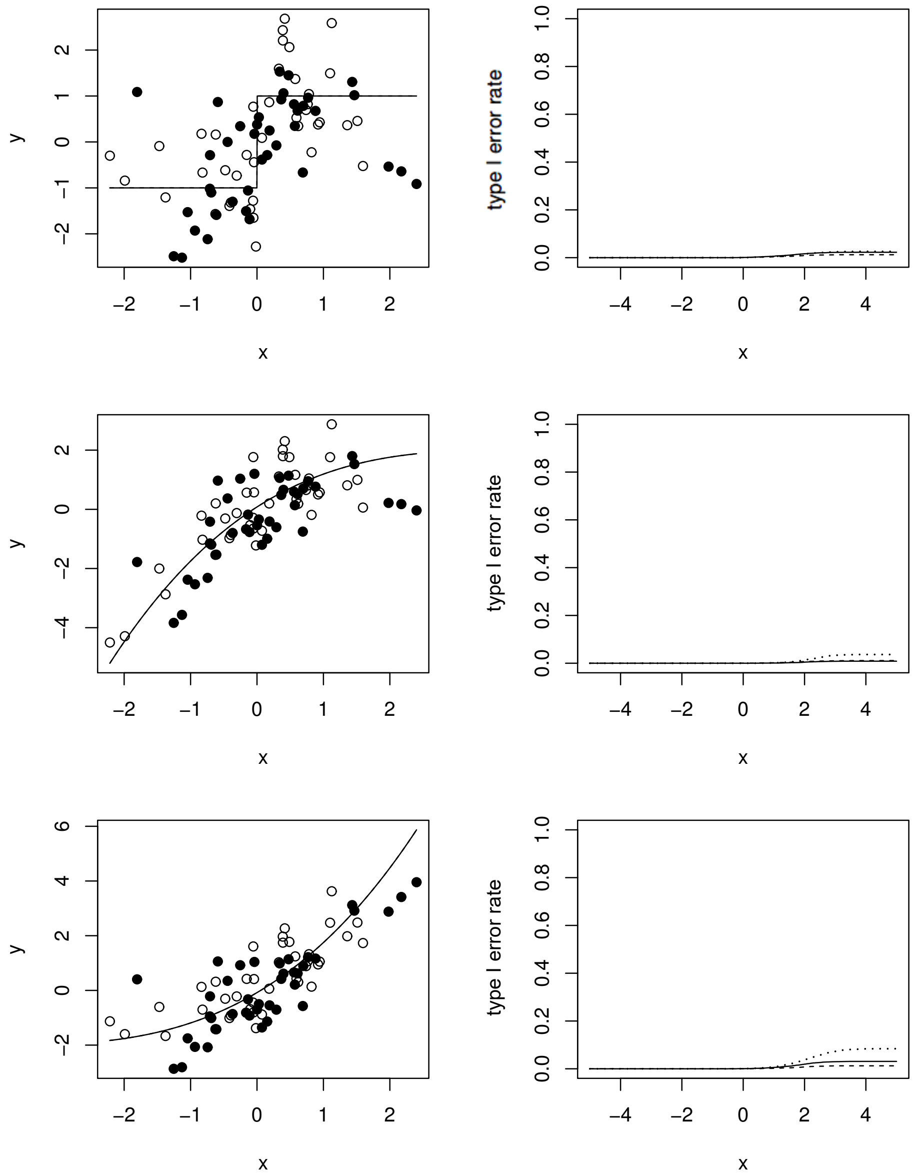

Figure A.1: Simulation models and example data sets and simulation results models with non-linear prognostic biomarker effects given in Table 2 of the paper. Left hand panels show simulation models and one simulated data set for $t = 0$ (dashed line gives expected values and hollow points give simulated values) and $t = 1$ (solid line gives expected values and solid points give simulated values). Right hand panels show simulated probability from 10,000 simulations per scenario of rejecting H_k plotted against x_k for tests as in Stallard (2023) (dashed lline) and using Z_k (dotted line) and Z_k^* (solid line). Probabilities shown are type I error rates.

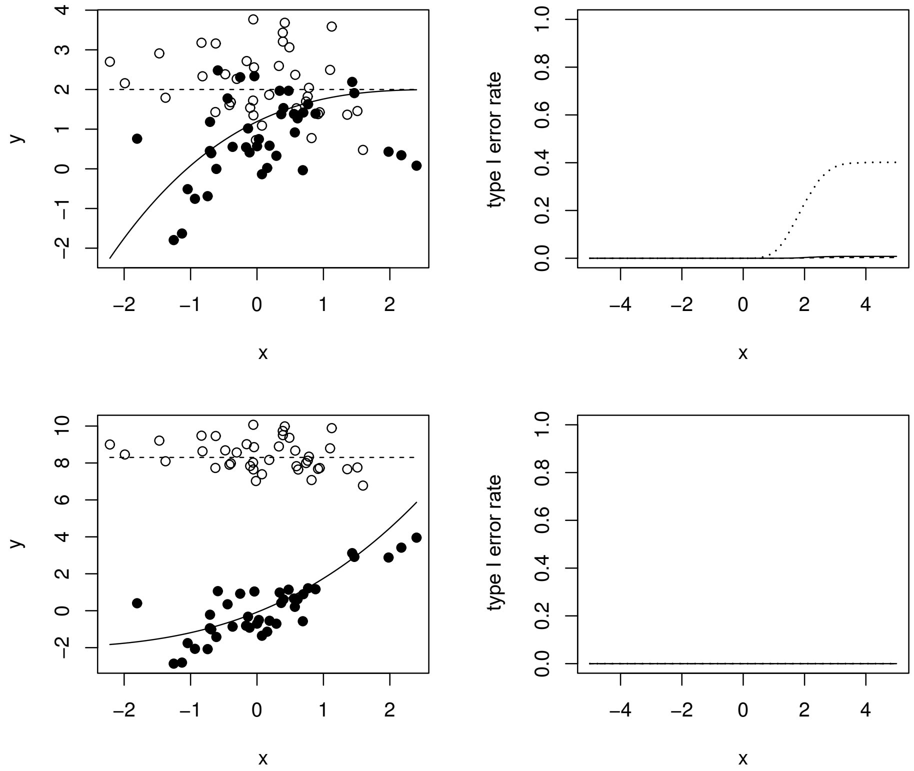

Figure A.2: Simulation models and example data sets and simulation results models with non-linear predictive biomarker effects with no positive treatment effect given in Table 2 of the paper. Left hand panels show simulation models and one simulated data set for $t = 0$ (dashed line gives expected values and hollow points give simulated values) and $t = 1$ (solid line gives expected values and solid points give simulated values). Right hand panels show simulated probability from 10,000 simulations per scenario of rejecting H_k plotted against x_k for tests as in Stallard (2023) (dashed lline) and using Z_k (dotted line) and Z_k^* (solid line). Probabilities shown are type I error rates.

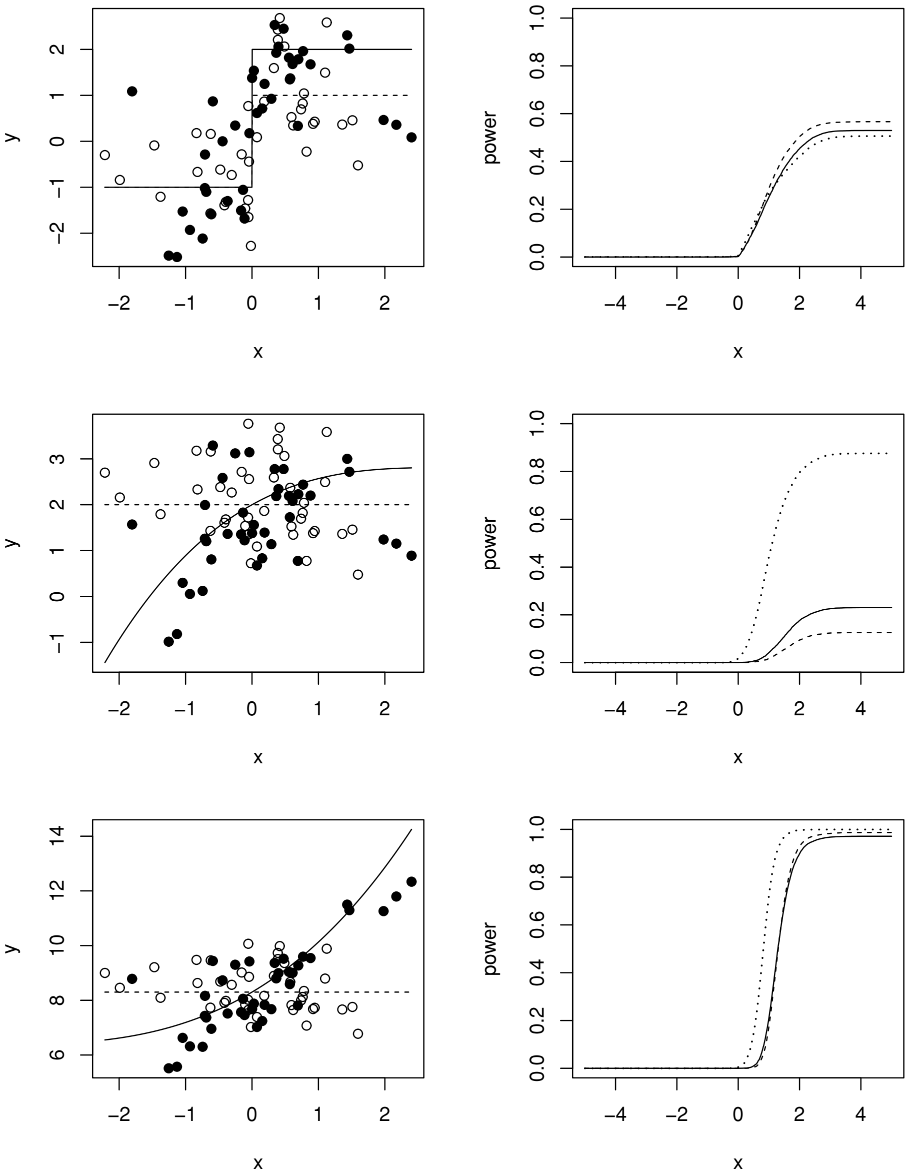

Figure A.3: Simulation models and example data sets and simulation results models with non-linear predictive biomarker effects with positive treatment effects for $x > 0$ given in Table 2 of the paper. Left hand panels show simulation models and one simulated data set for $t = 0$ (dashed line gives expected values and hollow points give simulated values) and $t = 1$ (solid line gives expected values and solid points give simulated values). Right hand panels show simulated probability from 10,000 simulations per scenario of rejecting H_k plotted against x_k for tests as in Stallard (2023) (dashed lline) and using Z_k (dotted line) and Z_k^* (solid line). Probabilities shown are type I error rates for $x \leq 0$ and power for $x > 0$.
